# Implementation of Kinetic and Kinematic Variables in Ergonomic Risk Assessment Using Motion Capture Simulation: A Review

**DOI:** 10.3390/ijerph18168342

**Published:** 2021-08-06

**Authors:** Muhamad Nurul Hisyam Yunus, Mohd Hafiidz Jaafar, Ahmad Sufril Azlan Mohamed, Nur Zaidi Azraai, Md. Sohrab Hossain

**Affiliations:** 1 School of Industrial Technology, Universiti Sains Malaysia, USM, Penang 11800, Malaysia; hisyam_yunus@student.usm.my; 2 National Poison Centre, Universiti Sains Malaysia, USM, Penang 11800, Malaysia; 3 School of Computer Sciences, Universiti Sains Malaysia, USM, Penang 11800, Malaysia; sufril@usm.my; 4 School of the Arts, Universiti Sains Malaysia, USM, Penang 11800, Malaysia; nurzaidi@usm.my

**Keywords:** kinetic variables, kinematic variables, ergonomic risk assessment, motion capture

## Abstract

Work-related musculoskeletal disorders (WMSDs) are among the most common disorders in any work sector and industry. Ergonomic risk assessment can reduce the risk of WMSDs. Motion capture that can provide accurate and real-time quantitative data has been widely used as a tool for ergonomic risk assessment. However, most ergonomic risk assessments that use motion capture still depend on the traditional ergonomic risk assessment method, focusing on qualitative data. Therefore, this article aims to provide a view on the ergonomic risk assessment and apply current motion capture technology to understand classical mechanics of physics that include velocity, acceleration, force, and momentum in ergonomic risk assessment. This review suggests that using motion capture technologies with kinetic and kinematic variables, such as velocity, acceleration, and force, can help avoid inconsistency and develop more reliable results in ergonomic risk assessment. Most studies related to the physical measurement conducted with motion capture prefer to use non-optical motion capture because it is a low-cost system and simple experimental setup. However, the present review reveals that optical motion capture can provide more accurate data.

## 1. Introduction

**Figure 1:**
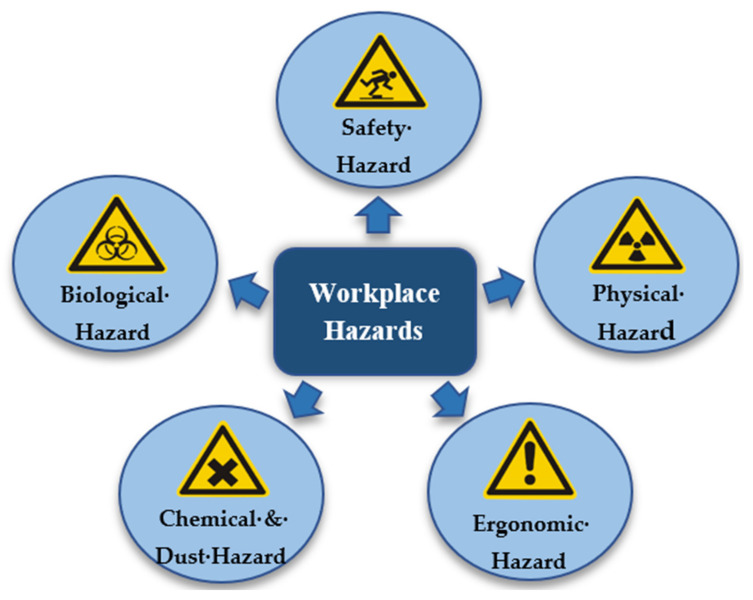
The five main hazards involve in workplaces.

All job sectors and industry sectors have various risks that can cause accidents and injuries. The study on the safety and health of industrial activities is critical to providing a better atmosphere in the workplace. Figure 1 shows five main categories of hazards related to the workplace according to occupational safety and health standards.

**Figure 2:**
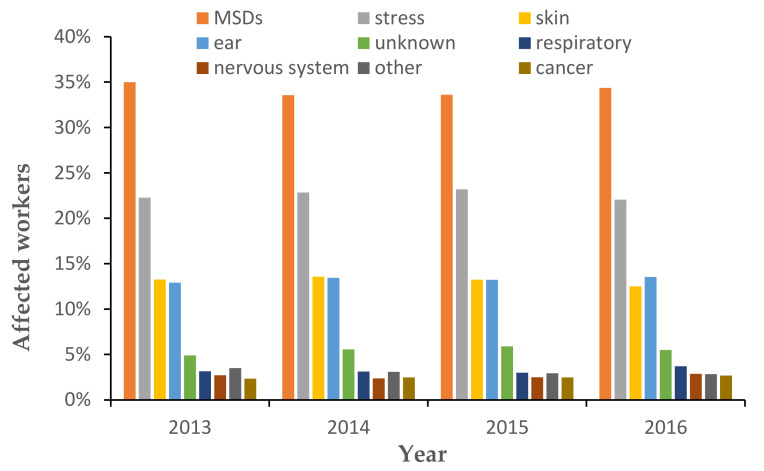
Number of workers affected by diseases reported by the group in Europe [8].

Ergonomics is the study to design workstations, work practices, and workflows for the worker in order to reduce risk factors such as sprains, strains, and cumulative trauma disorders [1]. The objective of an ergonomics assessment is to determine the level of risk for work-related tasks [1]. Unergonomic working conditions can contribute to discomfort and fatigue, leading to muscle, tendon, ligament, nerve, and blood vessel injury. This group of injuries is known as musculoskeletal disorders [2]. Work-related musculoskeletal disorder is the most common injury in the workplace [3]. Common risk factors for work-related musculoskeletal disorders are task frequency, manual material handling, load weight, awkward posture, vibration, flexion angle, and extension angle [4,5,6]. This disorder will cause pain, injury, illness, negative economic impact, reduced work performance, and decreased productivity [2,7]. Figure 2 shows the number of workers affected by diseases reported by the group in Europe from 2013 to 2015 [8]. It was also observed that musculoskeletal disorder constitutes a major work health issue compared to other work-related diseases. Therefore, it is crucial to design an ergonomic workstation to reduce musculoskeletal disorder.

Rapid Upper Limb Assessment (RULA), Rapid Entire Body Assessment (REBA), Agricultural Upper-Limb assessment (AULA), and Ovako Working Posture Analysis System (OWAS) are among many ergonomic assessment tools to evaluate ergonomic risk factors [7,9]. These tools have been used widely in the industrial sector. Most ergonomic risk assessment tools focus on the qualitative view to describe the risk level of motion and posture. The aim of this review is to assess the technology of motion capture that is suitable to evaluate ergonomic risk assessment in various industries. Motion capture technology includes optical and non-optical motion capture. The suggestions of this review would provide a view on the ergonomic risk assessment method and apply the current technology motion capture with the understanding of classical mechanics of physics that include velocity, acceleration, force, and momentum in ergonomic risk assessment.

## 2. Motion Capture Simulation

The standard way to assess ergonomic risk is by using motion capture technology [10,11,12,13,14]. Table 1 shows the advantage and limitations of motion capture systems in determining ergonomic risk assessment. Motion capture is the process of tracking the motion of a subject digitally. An ergonomic risk assessment using motion capture comes under the category of direct measurement methods, which use depth sensors to capture human motion [10]. These methods are believed to be the most accurate for providing reliable data to evaluate the ergonomic risk [5,15]. Motion capture is a powerful tool that can be used in many applications for human motion analysis [16]. Motion capture can provide data consisting of a skeleton diagram, body angle deviation, body velocity, and body acceleration [14,17,18]. Several researchers have exploited motion capture technology to evaluate recent ergonomic risk assessment tools such as RULA and REBA [14,18].

**Figure 3:**
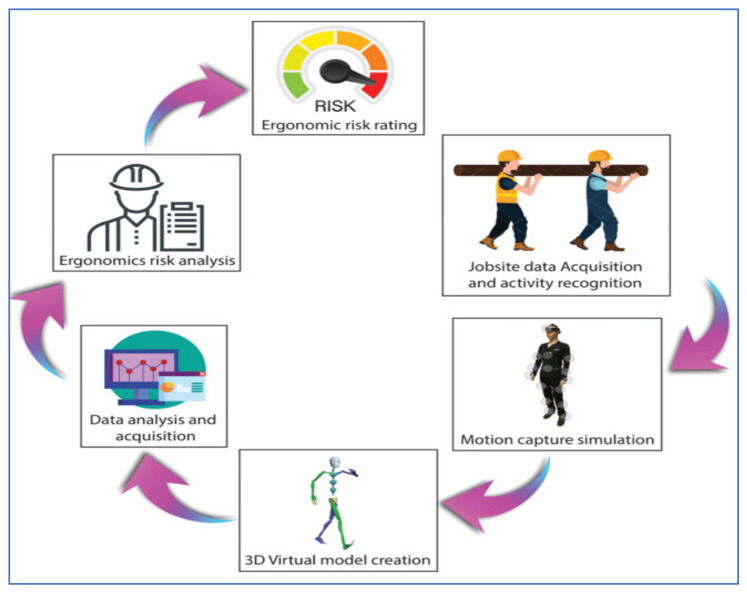
Workflow for the ergonomics risk assessment using motion capture technology.

The workflow for an ergonomic risk assessment using motion capture technology is shown in Figure 3. The use of motion capture simulation in assessing ergonomic risk is a remarkable improvement because this simulation can provide detailed and accurate human motion data [35]. However, a recent study that used motion capture technologies in ergonomic risk assessment is still based on the ergonomic assessment methods [23,36]. It would be helpful if the data from motion capture technologies such as body velocity and body acceleration were optimally used to analyze human motion. The added value of body velocity and body acceleration can contribute a more reliable result in ergonomic risk assessment. Inconsistency and biased judgment can be avoided with the use of accurate quantitative data on the evaluation from motion capture simulation. The assessment can also be carried out without the presence of an advanced ergonomist or physiologist. Therefore, there is a need to develop a complete method that uses a quantitative technique for better understanding the ergonomic risk factor and developing preventative steps [16,37]. Unfortunately, high accuracy motion capture systems, such as the Vicon tracking system, are not portable and are costly [37]. However, low-cost motion capture systems, such as Kinect, cannot provide reliable and accurate data for biomechanical analysis [38].

**Figure 4:**
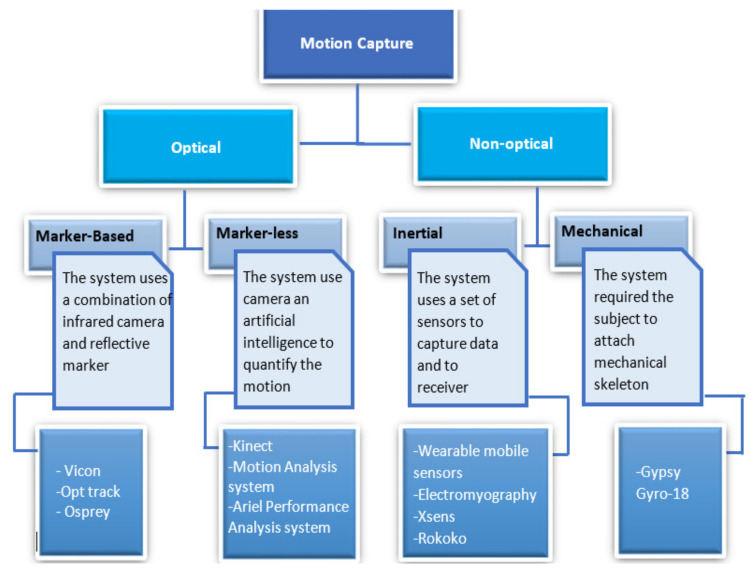
Optical and Non-optical motion capture simulation for ergonomics risk assessment.

Motion capture can be categorized into optical and non-optical methods [31]. Figure 4 shows the difference between optical and non-optical motion capture simulation for ergonomic risk assessment [10,31,39]. An optical motion capture system is a camera-based method, while the non-optical motion capture system is tracking motion from the relative position of different segments [31]. However, optical motion capture is suitable to assess aggressive and dynamic human activities [10,40]. This is because optical motion capture uses cameras, and the number of cameras and the frame rate can, therefore, be adjusted based on the motion capture requirements [10]. Currently, there are two types of optical motion capture used for biomechanical studies, which are marker-based and markerless motion capture [39,40]. Conversely, non-optical motion capture is tracking the motion without a visual field. It enables assessments to be performed in areas where the visibility is impaired or impossible [10]. A well-known non-optical motion capture system is an inertial measurement unit (IMU) that uses wearable sensors such as accelerometers, gyroscopes, and magnetometers on the body to capture the motion [15,28,31]. Motion capture is beneficial in a number of research fields related to biomechanics, but the accuracy of the system must be assured [41]. Both motion capture systems have their advantages and disadvantages based on the purpose of their use. Humadi et al. [42] determined the reliability and accuracy of non-optical and optical motion capture technologies using a wearable technology and a markerless optical technology, respectively, for different manual materials handling tasks. The study reported that the non-optical motion capture technology is more suitable than markerless optical technology in ergonomics risk assessment. Brunner et al. [43] determined ergonomic risk assessment in occupational practice using marker-based (Vicon Bonita) and markerless (Microsoft Kinect V2) optical motion capture systems, wherein the captured working postures were evaluated by analyzing the angles of different body segments. The study reported that markerless motion capture measured greater values than the marker-based motion capture in estimating potential health risk while conducting postural ergonomic risk assessments. However, Fletcher et al. [10] evaluated the ergonomic risk assessment of manufacturing work using the inertial non-optical motion capture. Their study reported that the non-optical motion capture method could better record motion data suitable for postural analysis from within an occluded workplace.

## 3. Ergonomic Risk Assessment

Ergonomic risk assessment is an important task in evaluating risk activities in the workplace [1,44]. Ergonomic risk assessment supports provision of a healthy work environment and the design of a workplace for optimum work performance [1]. Table 2 shows the advantages of ergonomic risk assessment in a work sector. 

The physical approach is the most common method that ergonomists have used in ergonomic risk assessment, and it can be divided into self-report, systematic observational, and direct measurement [7,54]. Each method has its own advantages and disadvantages. This present study reviewed the three ergonomic risk assessment methods involved in the physical approach. The self-report method uses a set of questionnaires and surveys to quantify and assess the discomfort severity experienced by the worker. This method is useful to study the experience of workers as the discomfort cannot be observed and measured directly [52]. Most musculoskeletal disorders begin with discomfort and stress experienced by workers. The Nordic Musculoskeletal Questionnaire and Cornell Musculoskeletal Discomfort Questionnaire are examples of tools based on this method [45,46]. The advantage of this method is that no technical equipment is needed, making it relatively inexpensive and easy, with the data and input directly from the subject [47]. This approach is also suitable to be conducted on a large scale of issues [48]. In a recent study, Huang et al. [44] developed a method for assessing WMSDs in the workspace using a wearable inertial sensor-based automated system to overcome the limitations of traditional WMSDs risk assessment methods. The study reported that the wearable inertial sensor-based automated system has the potential to be used for WMSDs risk assessments of workers when performing tasks in a workspace. Kim et al. [57] employed a new open-source human post estimation technology (OpenPose) for computing RULA/REBA scores and joint angles to determine ergonomic postural assessment. It was found that OpenPose has the potential to be utilized as a promising technology to determine semi-automatic ergonomic postural assessments and to measure joint angles in the non-ideal condition of a real workspace.

Observational methods provide ergonomists with postural evaluation tools to assess the ergonomic risk. This method can be used to analyze various occupational tasks and the workplace. Rapid Upper Limb Assessment and Rapid Entire Body Assessment are the preeminent and easiest tools for rapid ergonomic risk assessment in observational methods to evaluate work-posture [49,58]. RULA provides the level of postural load for the upper limb and REBA for the entire body [53,59]. Other ergonomic assessments, such as the Quick Exposure Checklist and the Strain Index, are also used for ergonomic risk assessment [50,55]. The advantages of these tools are that they can be conducted by inexperienced users as these tools are easy to learn and quick to use [51,52]. These ergonomic risk assessment tools also give an immediate result and mainly cover whole body posture to adequately assess work-related musculoskeletal disorder risk [7,53].

Direct measurement methods have a similar objective to observational methods but with different basic approaches [29]. This method is an instrument-based method that collects the information through the sensor attached to the body of the subject [54]. Generally, the direct measurement method is preferred for the research context [52]. This approach requires software, sensor, and data logger to obtain the quantitative data, for example, Lumbar Motion Monitor (LMM), force sensor, and electromyography [55,56]. The advantage of this method is it offers high accuracy of posture assessment for musculoskeletal studies [52]. This method is also useful to quantify the quantitative data, such as force exerted, which cannot be assessed from observation. [22].

Despite the advantages listed, some researchers still raised an issue about the frailty and the limitations among current ergonomic risk assessment methods [56,60,61,62]. The question about accuracy and consistency in ergonomic risk assessment methods is often discussed academically. Studies on the validity and reliability of the Nordic Musculoskeletal Questionnaire have found that there is very little information related to psychometric properties [47,48]. Current ergonomic risk assessment tools only focus on the posture of the movement without analyzing the motion of the movement. RULA, REBA, and many other ergonomic risk assessment methods rely upon direct observation [63]. This method of ergonomic risk assessment needs a certified professional ergonomist involved to evaluate the assessment, and potentially the rate of ergonomic risk is biased due to subjective judgment [64,65]. The assessment result among all observational ergonomic risk assessment methods is inconsistent because of differing basic approaches [65]. Most ergonomic assessments claimed that the method could include the variable of force to evaluate the risk from the motion. The fact is force means the product of acceleration and mass or load. Unfortunately, the body acceleration is neglected during the assessment.

## 4. Kinetic and Kinematic Variable

To assess the ergonomic risk from analyzing the motion, the classical mechanics of physics approach is needed. There are three fundamental laws in classical mechanics, such as [66,67]:

(a) Newton’s first law: object will remain at rest or constant velocity unless an external force acts on it;
(b) Newton’s second law: the force is equal to the product of mass and acceleration; and
(c) Newton’s third law: when a body exerts a force on another body, the body will have equal force with the first body.

This shows that if force is used to describe the motion, then body velocity and body acceleration also need to be discussed because velocity, acceleration, and force correlate. Velocity is the displacement over time, acceleration is the derivative of velocity, and force is the product of the derivative of velocity and mass. Equations (1)–(3) describe the relation of velocity, acceleration, and force.

(1) $$v\left(t\right)=\frac{dx}{dt}$$

(2) $$a\left(t\right)=\frac{d^{2}x}{dt^{2}}=\frac{v\left(t\right)}{dt}$$

(3) $$f=m\frac{v\left(t\right)}{dt}=m\;a\left(t\right)$$

where $v\left(t\right)$ is velocity, $a\left(t\right)\;$ is acceleration, f  is force, dx  is displacement, dt  is the time taken, and m is mass. Research using the electromyography approach has shown that any muscle activity, such as during lifting, correlates with the physical measure consisting of velocity, acceleration, and force [68]. This indicates that input data related to physical measures are necessary for evaluating the ergonomic risk assessment. Therefore, using quantitative data in the ergonomic risk assessment is important to reduce the risk of musculoskeletal disorder [17]. With accessibility to technology such as motion capture, which can provide data on velocity, acceleration, and force, assessing ergonomic risk by evaluating this variable from body motion is now possible. Table 3 summarizes the recent studies related to the kinematic and kinematic variable by using motion capture.

### 4.1. Velocity and Acceleration

The main factor contributing to musculoskeletal disorder is the frequency, magnitude, and time taken of musculoskeletal load exerted on the join of the subject [17]. Velocity and acceleration are the correct terms to discuss work time is taken, pace, magnitude, and frequency. The difference between speed and velocity is that velocity consists of magnitude and direction, while speed is only a magnitude. Data for body velocity and acceleration can evaluate the pace of the movement [22]. Reducing processing time is an appropriate solution to cut costs. This causes the worker to move rapidly or work at a high pace [34]. Doing a job at a fast pace does not mean it is right, but it can turn out to be a risk factor. The previous study has also proved that a slow working pace is more hazardous than a fast-working pace [72]. This shows that it is essential to do work at an optimal working pace. The data for body velocity and acceleration can be used to compare the work pace of experienced and novice workers [19]. This method can be a benchmark to develop the optimal work pace. Therefore, to determine the optimal work pace in manual handling tasks, occupational risk factors, and work position standards that follow the ergonomic intervention are very important [2]. They can increase productivity and reduce the risk of musculoskeletal injury.

Recent ergonomic risk assessment tools, such as REBA and RULA, only include load to evaluate the body posture. Working pace can affect muscle temporal recruitment pattern while lifting, such as triceps, biceps, deltoid, and trapezius, but load or weight during lifting did not affect temporal muscle recruitment pattern [73]. Waddell and Amazeen [30] found that the perceived heaviness during lifting tasks correlates with muscle activity and lifting speed. The lifting speed has also been found to be affected by peak compressive force [10]. Medium pace during the initial lifting is very beneficial to avoid jerky or awkward movement and produce less back compressive force [72]. Hence, to assess the ergonomic risk of tasks that deal with the load, the body velocity and acceleration also need to be considered to achieve reliable results.

Other than evaluating the work pace, data for velocity and acceleration from the motion capture can be used to improve the workstation layout [18]. Motion capture can map the movement of the subject with detail on the velocity and displacement [18,19,74]. This can help to reduce unnecessary action during walking, for example walking throughout the working station. Other than for ergonomic risk assessment, this data can be used to compare the movement of experienced workers and novice workers, which can improve work performance [19]. This method can also verify the effect of environmental changes to the behavior of the subject in terms of their work performance and work [23].

The data for initial acceleration from the body motion have a high correlation with work-related musculoskeletal disorder [34]. From Equation (2), velocity and acceleration input data are closely related to each other. When the body velocity increases, the body is accelerating; while the body velocity decreases, it indicates that the body is decelerating. In another case, if the body has a constant velocity, it means the body acceleration is zero. The movement shows much acceleration, and deceleration indicates that the movement is vigorous and dynamic. The data for body acceleration can also be used to determine the range of motion or the angle of deviation [28]. Angle deviation is the degree of bend from the neutral posture where all body parts are in a state of minimal effort, physical stress on bones, muscles, nerves, or tendons [28]. When the body velocity is increasing rapidly, which means that acceleration exists, the body angle deviation and the range of motion are higher [17,22]. This shows that mathematically body acceleration could predict the trunk and shoulder flexion with an accurate angle [28]. Inertial motion capture systems such as wearable accelerometers use this method to predict the angle of deviation [28,31,67]. However, the accuracy is low for dynamic movement [31,62]. Equation (8) describes the formula for predicting the angle of trunk and shoulder flexion [28].

(4) $$P_{x}=cos^{-1}\left(\frac{P_{x}\_acc\_max-P_{90}\_acc\_max}{P_{1}\_acc\_mac-P_{90}\_acc\_max}\right)$$

where $P_{x}$ is the total angle of deviation for selected postures, $P_{x}\_acc\_max\;$ is the acceleration for the selected postures, $P_{1}\_acc\_max$ is the initial acceleration for the selected posture, and $P_{90}\_acc\_max$ is the acceleration for a 90° selected posture. The acceleration for initial and 90° is pre-recorded before the actual observation with the same work pace. Generally, from the ergonomic risk assessment perspective, the body angle deviation determines the awkward posture [28]. Awkward posture and overexertion are factors that can contribute to work-related musculoskeletal disorder [26,75]. However, previous methods of ergonomic risk assessment did not discuss this matter in detail [26]. In physical terms, they describe inertia awkwardly. In addition, it uses data on body acceleration to describe inertia. Awkward posture happens when the acceleration is high. Therefore, the body acceleration from the movement must be lower to achieve an ergonomic movement.

The body velocity and acceleration data can be obtained through optical motion capture or non-optical motion capture such as an accelerometer [15,64,67]. Equations (4)–(6) describes the calculation for velocity collected through optical motion capture if the frame rate is set to 9 [56].

(5) $$v\left(y_{i}\right)=\frac{\left(FR\right)\times \left(y\left(i+4\right)-y\left(i-4\right)\right)}{8}$$

(6) $$v\left(z_{i}\right)=\frac{\left(FR\right)\times \left(z\left(i+4\right)-z\left(i-4\right)\right)}{8}$$

where *FR* is the frame rate, and *i* is the number of frames. The resultant velocity, $R_{v}$ can be determined as:

(7) $$R_{v}=\sqrt{v{\left(x_{i}\right)}^{2}+v{\left(y_{i}\right)}^{2}+v{\left(z_{i}\right)}^{2}}$$

The below equations describes the calculation for body acceleration, which are the derivative from Equations (4)–(6) [62].

(8) $$a\left(x_{i}\right)=\frac{d}{dt}v\left(x_{i}\right)=\frac{FR^{2}\times \left(x\left(i+4\right)-2x\left(i\right)+x\left(i-4\right)\right)}{16}$$

(9) $$a\left(y_{i}\right)=\frac{d}{dt}v\left(y_{i}\right)=\frac{FR^{2}\times \left(y\left(i+4\right)-2y\left(i\right)+y\left(i-4\right)\right)}{16}$$

(10) $$a\left(z_{i}\right)=\frac{d}{dt}v\left(z_{i}\right)=\frac{FR^{2}\times \left(z\left(i+4\right)-2z\left(i\right)+z\left(i-4\right)\right)}{16}$$

The resultant acceleration can be determined as:

(11) $$R_{a}=\sqrt{a{\left(x_{i}\right)}^{2}+a{\left(y_{i}\right)}^{2}+a{\left(z_{i}\right)}^{2}}$$

### 4.2. Force

Body force is generated by the acceleration of the body part during the movement [10]. From Equation (3), the data for body acceleration can determine the force exerted by the body during the movement. This indicates that the force applied by muscle correlates with body acceleration [29]. Direct measurement of the force exerted during physical tasks can effectively reduce the risk of work-related musculoskeletal disorder [17,20,29]. A musculoskeletal disorder can occur when a normal force is exerted on weak tissues or when high force is exerted on normal tissues [20]. When a body holds an object, muscle activity is required to resist gravity force, which shows the correlation of muscle activity with force during muscle flexion and contraction [30]. During manual handling tasks such as lifting and carrying the load, the magnitude of the force exerted on the muscle’s joint directly impacts the magnitude of joint reaction [20]. Thus, the evaluation of the body forces during the movement is important in determining the ergonomic risk.

There are many ways to predict the force exerted by the body. The body force can be calculated using Baltimore therapeutic equipment to simulate work activities related to pushing and pulling [29]. The data output from this simulator is in watt P. The approximation of body net force exerted is achieved by using the equation below [29].

(12) $$F=\frac{P\times t}{d}$$

where P is watt in kgm^2^/s^−3^, *t* is time in second (s), and *d* is displacement in meter (m). In this study, the time and displacement were measured through observation. From Equation (12), it can be assessed that time over displacement is the data for body velocity. Velocity can be measured using optical motion capture that gives more accurate data than when measured through direct observation. Additionally, another approach to predict body force is by using an electromyogram (EMG), a non-optical motion capture system, where the force predicted by electromyography is considered as the force exerted by muscle or muscle activity [23,66,68]. This method requires the subject to wear a wired sensor directly to their skin, which is somewhat difficult and intrusive [63]. The validity of the predicted force obtained from this method is still in question. This is because the force is the product of mass and the rate of change of velocity, and the force predicted by this system neglects the mass during the observation. EMG may reflect the demand for muscle tissue, higher demand being associated with high force applied [69]. Another well-known method for obtaining body force is by using a force plate and foot plant combined with motion capture technologies [32,33,41,76]. However, this method only can obtain the body force for certain tasks. This shows that motion capture can help to approximate the body force applied from the body movement. Table 4 summarizes the contribution of kinetic and kinematic variables to the ergonomic risk assessment.

## 5. Conclusions

This review has highlighted how current technology can analyze body motion using kinetic and kinematic variables with high accuracy. These variables, such as velocity, acceleration, and force, can give an added value in ergonomic risk assessment to achieve more reliable results. Evaluation of ergonomic risk assessment through quantitative data can avoid inconsistency and biased judgment in the assessment. The review was conducted to develop a complete method of ergonomic risk assessment, wherein the evaluation of kinetic and kinematic variables is very important. The more precise result achieved from the ergonomic risk assessment can increase accuracy in identifying the risk level of work tasks. As a result, the risk of work-related musculoskeletal disorder can be reduced. It can be postulated that the most recent studies that focus on the kinetic and kinematic variables used non-optical motion capture rather than optical motion capture. This is because optical motion capture is a very expensive and complicated experimental setup. However, the present review reveals that optical motion capture is more accurate than non-optical motion capture.

## Figures and Tables

**Table 1:** Advantages and limitations of motion capture systems in determining ergonomic risk assessment.

Motion Capture	Type	Data	Advantage	Disadvantage	References
Optical	Markerless	- Time taken - Displacement - Body Velocity - Body Acceleration	- Low cost - Portable - Real-time result - Simple start-up	- Less accurate - Magnetic interference noise	[17,18,19,20,21]
	Marker Based	- Time taken - Displacement - Body Velocity - Body acceleration - Body angle deviation - Body posture	- Accurate - Fast - Automatic tool analysis - Real time result	- Costly - Complex tool - Not Portable	[22,23,24,25]
Non-Optical	Pressure sensor	- Pressure - Force	- Low cost - Simple experiment set-up	- Limited to relatively low pressure	[26,27]
	Inertial	- Electrical pulse (Muscle activity) - Body acceleration - Body velocity	- Potable - Low cost	- Less accurate - Can cause discomfort to the subject - Magnetic interference noise	[28,29,30,31]
	Force plate	- Force	- Portable		[32,33]
	Mechanical	- Time taken - Body velocity - Body acceleration - Body posture	- High Accuracy	- Cause discomfort to subject - Heavy	[34]

**Table 2:** The advantage of ergonomics risk assessment methods.

Ergonomic Assessment Method	Tools	Advantage	References
Self-report	Nordic Musculoskeletal Questionnaire (NMQ) Cornell Musculoskeletal Discomfort Questionnaire (CMDQ)	No technical equipment needed Inexpensive Suitable for large scale assessment	[45,46,47,48]
Observational	Rapid upper-limb assessment (RULA) Rapid entire body assessment (REBA) Quick exposure checklist (QEC) Strain index	Easy to use Quick assessment Cover whole body posture	[49,50,51,52,53]
Direct measurement	Lumbar Motion Monitor (LMM) Force sensor Electromyography	High accuracy Able to quantify quantitative data Suitable for research context	[29,54,55,56]

**Table 3:** Summary of recent studies related to kinetic and kinematic variables by using motion capture.

Data Input	Motion Capture Type	System	Research Scope/Finding	References
Velocity	Optical	Microsoft Kinect V2	Evaluate the cycle time of worker in the set-up workstation	[18]
		Kinect based	Compare the martial art performance (Silat) between novice and experienced performer	[19]
		Optitrack	Evaluate the kinematic data of shoulder and elbow during walking with different pace	[22]
Acceleration	Optical	Ipi soft Motion capture	The maximum back compressive force produced during high acceleration and angle of trunk flexion	[17]
		Northern Digital Optotrak 3020 motion tracking system	Perceived heaviness is the function of ratio of muscle activity to acceleration	[30]
	Non-Optical	Wearable accelerometer	Predict the angle of deviation for shoulder and trunk flexion using the angular acceleration	[28]
		Wearable accelerometer	Proposed low-cost wearable inertial sensor to track the upper body movement	[69]
		Wearable accelerometer	The proposed wearable sensor is potentially acceptable for slow tasks to predict the trunk flexion	[64]
		OpenGo system (Moticon)	Evaluate the risk of overexertion based on acceleration and pressure data	[26]
Force	Optical	Ariel performance analysis system (APAS)	The musculoskeletal injury happened when normal forces are exerted to abnormally weak tissues or when high forces are exerted to normal tissues	[20]
	Non-Optical	Electromyogram (EMG)	The higher the trunk flexion angle, the higher the compression force	[70]
		Electromyogram (EMG)	Anterior deltoid and upper trapezius are under high demand during load transfer tasks for people with lower back pain and spinal cord injury	[71]
		Baltimore therapeutic equipment (BTE)	Data collected during the study using accelerometer sensor has a correlation with force applied by muscle	[29]
		Force plate	Calculate the ground reaction forces and moment from the walking task with different pace	[41]

**Table 4:** Summary for the contribution of the kinetic and kinematic variables to the ergonomic risk assessment.

Variable	Contribution
Velocity	Identify the suitable pace during working. Evaluate working movement and posture. Design workstation layout.
Acceleration	Identify the angle of deviation for every joint. Determine the awkward posture. Recognize the repetitive movement during work. Assess the rate of change of velocity. Calculate the force exerted from work.
Force	Determine the optimal load carried by worker. Calculate the momentum and work done. Evaluate the muscle activity. Evaluate the ergonomic risk.

## Data Availability

Not applicable.
